# Idiosyncratic Drug-Induced Liver Injury in a Healthy Patient following PCSK9-Inhibitor Injection

**DOI:** 10.1155/2024/5556907

**Published:** 2024-01-11

**Authors:** B.-O. Stüben, D. P. Hoyer, S. Radunz, F. Saner, H. Schmidt, H. A. Baba, J. W. Treckmann, L. I. Mazilescu

**Affiliations:** ^1^Department of General, Visceral and Transplant Surgery, Medical Center University Duisburg-Essen, Essen 45147, Germany; ^2^Department of Internal Medicine, Medical Center University Duisburg-Essen, Essen 45147, Germany; ^3^Institute of Pathology, Medical Center University Duisburg-Essen, Essen 45147, Germany

## Abstract

**Background:**

Acute liver injury is a life-threatening condition with disparate aetiology. Swift and adequate interdisciplinary treatment is essential to assure the best possible outcomes in these patients. Investigations to identify the cause of the condition and the implementation of quick and appropriate treatment can be lifesaving. *Case Presentation*. In October 2022, an otherwise healthy 66-year-old male presented at the University Hospital Essen with acute liver injury following an inclisiran injection for hypercholesterinaemia. Four weeks following admission, the patient fully recovered after initially receiving short-term cortisol therapy and open albumin (OPAL) dialysis, and the indices of liver, kidney, and coagulation function were normal at discharge.

**Conclusion:**

This is to our knowledge the first reported acute liver injury due to an inclisiran injection. Cortisol in combination with OPAL dialysis is an effective method for the treatment of acute liver injury caused by inclisiran injury, and in this case, it led to a near-complete reversal of the acute liver injury at the time of discharge.

## 1. Background

Proprotein convertase subtilisin/kexin type 9 (PCSK9) inhibition has emerged as a promising new therapeutic strategy to reduce low-density lipoprotein (LDL) levels [1–3]. Inclisiran is a novel, synthetic, small interfering RNA (siRNA) molecule inhibiting PCSK9 synthesis, which normally contributes to LDL-receptor degradation, leading to an increase in LDL-receptor density in hepatocytes. It received first approval in the United States in 2018 and in the European Union in December 2020 for use in adults for hypercholesterolaemia or mixed dyslipidaemia, intended for use in combination with a statin with the goal of lowering LDL levels [4]. Inclisiran is administered as a twice-yearly subcutaneous injection. Subcutaneous inclisiran injection has shown the same safety profile compared to placebo in the clinical trials performed [5–8]. Critically, in the phase III clinical trials (ORION-9, ORION-10, and ORION-11), the only adverse events were local reactions at the site of injection [6]. There was no evidence of kidney, liver, muscle, or platelet toxicity [9]. No overall differences in safety between younger patients and those ≥65 or ≥75 years in age were recorded.

We present the case of a 66-year-old patient who presented with an idiosyncratic drug-induced liver injury (DILI) three days after an inclisiran injection.

## 2. Case Presentation

A 66-year-old man presented with complaints of fatigue and decreased appetite for about three days and a sudden onset of jaundice. The patient had a history of familial LDL hypercholesterinaemia and no known liver disease. He had received a subcutaneous inclisiran injection due to myopathy under maximum statin dosages. The liver function tests (LFT) prior to the inclisiran injection were normal. Regarding comedication, the patient was under pantoprazole therapy at the standard dose for several years prior to presentation. At presentation, the patient had a BMI of 24.7 kg/m^2^. The patient was not diabetic and had no known liver disease.

On examination, the patient was in a stable cardiopulmonary state. There were no signs of hepatic encephalopathy (negative Reitan test). The patient had mucocutaneous jaundice and icteric conjunctivae without further skin manifestations of liver disease. Abdomen palpation showed no pathologies. Liver ultrasound showed a normal-sized liver without focal or diffuse lesions. There were no signs of a pre-existing fatty liver disease or nonalcoholic steatohepatitis (NASH). The gallbladder was normal without visible biliary stones. Perfusion of all hepatic vessels was normal. Computed tomography angiography showed a minimal amount of fluid between the liver and the gallbladder, discrete hepatic swelling, as well as attenuated lymph nodes in the duodenal hepatic ligament. Liver enzyme levels were significantly increased with a mild reduction in liver function. Other toxic or infectious causes of acute liver failure were ruled out.

**Figure 1:**
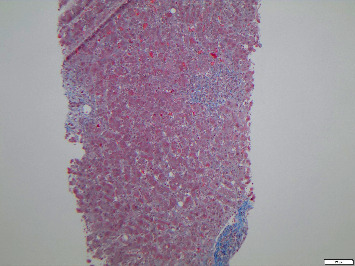
Modified Masson–Goldner elastica stain to visualize collagen (blue), nuclei (brown), and elastin (black). Portal fibrosis grade 1 is present.

**Figure 2:**
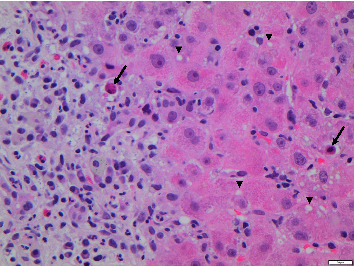
Hematoxylin and eosin staining; the portal infiltration consists of plasma cells, neutrophilic granulocytes, and eosinophilic granulocytes. Arrows indicate apoptotic bodies. Arrow heads indicate lipid vacuoles in hepatocytes. ^∗^Oil red staining to visualize lipids was not performed because the tissue was embedded in paraffin and snap-frozen material was not available.

Following admission to our hospital, the liver function tests (LFTs) and enzymes continued to rise for 11 consecutive days, with bilirubin peaking at 30.1 mg/dl (normal 1.1 mg/dl), INR (international normalized ratio) reaching a maximum of 1.39, ALT at 3428 U/l (normal 10–50 U/l), and AST at 3385 U/l (normal 10–50 U/l). An ultrasound-guided percutaneous liver biopsy was performed. Histopathology showed portal grade 1 fibrosis with significant portal and periportal hepatitis with the infiltration consisting of plasma cells, neutrophilic granulocytes, and eosinophilic granulocytes, as shown in Figures 1 and 2.

In the absence of significant spontaneous recovery, we started cortisol therapy on day 8 with an initial dose of 250 mg intravenous methylprednisolone. This was administered on three consecutive days, tapered, and withdrawn within seven days.

Following cortisol therapy, liver enzyme levels did not demonstrate a significant improvement.

Open albumin dialysis (OPAL) was first introduced in 2014. It is analogous to the molecular absorbent recirculating system (MARS) liver support system but with a greater filter surface, and a recent study comparing extracorporeal albumin dialysis showed that OPAL may provide higher reduction capabilities of liver solutes (i.e., bilirubin and ammonia) in comparison to MARS [10].

**Figure 3:**
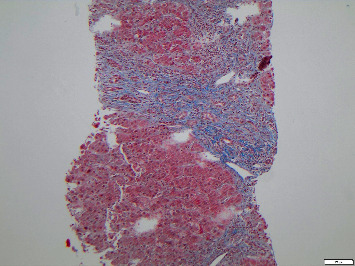
Modified Masson–Goldner elastica stain to visualize collagen (blue), nuclei (brown), and elastin (black). Periportal fibrosis grade 2 is present.

**Figure 4:**
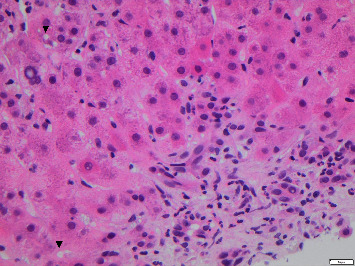
Hematoxylin and eosin stain: inflammation of the portal tract is reduced; neutrophils are still present, but the number of plasma cells and eosinophils has decreased. In this visual field, apoptotic bodies are not present. Arrow heads indicate lipid vacuoles. ^∗^Oil red staining to visualize lipids was not performed because the tissue was embedded in paraffin and snap-frozen material was not available.

OPAL was first performed on day 15 following admission. A total of 5 OPAL dialysis therapy sessions were completed, with a renewed liver biopsy carried out following the fifth treatment cycle. This showed a progression to grade 2 fibrosis but reduced periportal inflammation, with neutrophils still present, but the number of plasma cells and eosinophils having decreased. This is shown in Figures 3 and 4.

The patient was discharged after 41 days of treatment at our medical institution with markedly improved liver function (bilirubin 8.6 mg/dl, INR 1.06, ALT 570 U/l, AST 490 U/l). During the treatment duration, pantoprazole was discontinued but was resumed after improved lab results.

Follow-up of the patient following discharge showed a return to normal liver function, with the patient continuing to take pantoprazole.

**Figure 5:**
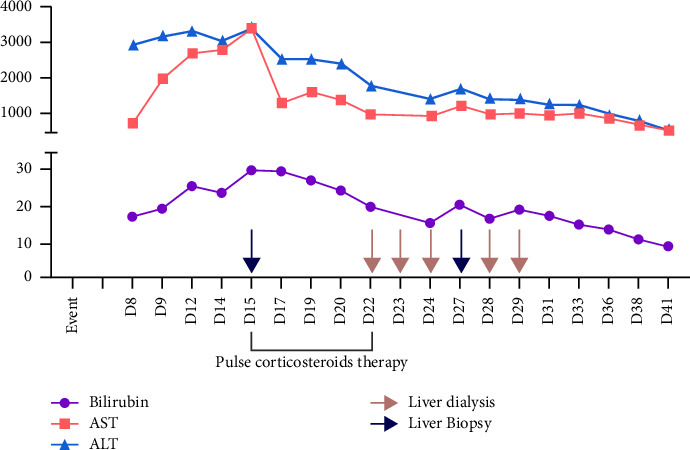
Liver enzyme and LFT levels over the course of the treatment with intervention times highlighted.

Figure 5 shows the laboratory trend during treatment and time of therapeutic interventions.

## 3. Discussion

siRNA therapeutics have been used for a variety of pathologies since they were first being introduced in 2018.

They have shown much promise and are on their way to becoming standard in pharmacotherapy. Inclisiran has shown potential for use aimed at a broad spectrum of patients with hypercholesterinaemia who fail to meet LDL targets even after maximal statin therapy. The trials for this drug have shown it to be safe with little to no side-effects and a mild adverse events profile (2).

We used the Roussel Uclaf Causality Assessment Method (RUCAM) scoring system for a causality assessment of the drug therapy and to define that idiosyncratic DILI was in fact present in this case. ALT levels were ≥5 times the upper limit of normal and ALP ≥2 times the upper limit of normal. Inclisiran was administered once at the recommended therapeutic dose. The worsening of the DILI under continued therapy could not be assessed as inclisiran is not administered daily but rather repeated after 3 months. According to the RUCAM scoring system, idiosyncratic DILI in this case is probable [11].

No existing liver biopsies or other liver-specific diagnostics had been performed prior to admission, so it cannot be stated with absolute certainty that the patient had an underlying liver disease prior to admission. Indeed, liver enzymes and function tests were in the normal range prior to admission.

The patient was under pantoprazole therapy at the standard dose for several years prior to presentation. A case report by Meunier et al. presented a case of DILI following 2 months of pantoprazole therapy, leading to chronic autoimmune liver injury with the presence of antibodies to nuclear antigens [12]. The patient required immunosuppressive agents for recovery. Another case of severe acute hepatitis in a patient one month after initiation of pantoprazole treatment with full recovery following discontinuation was reported by Sandig et al. in 2011 [13].

In this case, the histopathological specimen showed no signs of autoimmune liver injury, and all autoantibodies were negative. It could however be discussed that the progression from liver fibrosis grade 1 to 2 observed in this case may have been exacerbated by the concomitant pantoprazole therapy.

The combination of cortisol therapy and liver dialysis led to a reversal of the liver failure with an almost normal LFT at discharge from the hospital. The long-term implications of the drug-induced hepatitis remain unclear at this point.

The case has been reported by our institution to the relevant medical authorities in Germany, and a notice has been sent to the pharmaceutical company informing them about this event.

## Data Availability

All data stated in the article are available upon request from the corresponding author.
